# Gene Expression as a Guide for the Development of Novel Therapies in Hypertensive and Diabetic Kidney Disease

**DOI:** 10.3390/jcm15020696

**Published:** 2026-01-15

**Authors:** Maria Zaimi, Georgios Zagkotsis, Athanasios Kammenos, Eirini Grapsa, Smaragdi Marinaki, Eleni Frangou

**Affiliations:** 1 Department of Internal Medicine, Alexandra Hospital, 115 28 Athens, Greece; mariazaimi00@gmail.com; 2 Department of Nephrology, National and Kapodistrian University of Athens Medical School, Areteio Hospital, 115 28 Athens, Greece; 3 Department of Nephrology, Limassol General Hospital, State Health Services Organization, Limassol 4131, Cyprus; 4 Clinic of Nephrology and Renal Transplantation, National and Kapodistrian University of Athens Medical School, Laiko Hospital, 115 27 Athens, Greece; 5 Department of Basic and Clinical Science, University of Nicosia Medical School, Nicosia 2408, Cyprus

**Keywords:** diabetic nephropathy, hypertensive nephropathy, computational systems biology, gene expression, drug discovery and development, drug repurposing

## Abstract

**Background/Objectives**: Diabetes mellitus and hypertension are the first and second most common causes of chronic kidney disease, respectively. Despite improvements in elucidating the pathophysiology behind these diseases and the expansion of the therapeutic armamentarium, the knowledge about the implicated genes, epigenetics, and biological pathways is limited. **Methods**: We sought to define diabetic nephropathy-specific and hypertensive nephropathy-specific gene signatures in human glomeruli through computational systems biology approaches. **Results**: Gene expression data of human glomeruli from patients with diabetic kidney disease (DKD) and hypertensive nephropathy (HTN) were collected and compared to gene expression patterns from healthy kidneys. Pathways were identified with functional enrichment analysis of DEGs. Transcription factor enrichment analysis, protein–protein interaction network expansion, and kinase enrichment analysis were also performed. Finally, novel drugs and small-molecule compounds that may reverse the kidney-specific phenotype of these disorders have been identified. **Conclusions**: These data suggest putative expansion of the therapeutic armamentarium in DKD and HTN, underscoring that understanding the molecular mechanisms occurring within tissue in kidney diseases may guide personalized therapy.

## 1. Introduction

Diabetes mellitus and hypertension constitute the first and second leading causes of chronic kidney disease, respectively [1,2]. Forty percent of diabetic patients develop diabetic kidney disease (DKD), which is characterized by glomerulosclerosis, tubulointerstitial fibrosis, and inflammation [2], and persistent hypertension causes hypertensive nephropathy (HTN) via arteriolar damage, parenchymal injury, and tubulointerstitial fibrosis, accompanied by immune inflammation [3].

Although our understanding of the underlying pathophysiology of each disease has significantly advanced over the last few decades, and specific therapies, i.e., anti-diabetics against DM and anti-hypertensives against HTN, have been established, the molecular events at the glomerular and tubulointerstitial level characterizing these diseases remain elusive [4,5,6]. Current therapeutic options of DKD–including RAS inhibition, MRA, and SGLT2i–secondarily pose an anti-inflammatory action [7]. In a recent retrospective study, the triple therapy of RAS inhibitors, dapagliflozin, and finerenone was associated with a significant reduction in UPCR and UACR in patients with DKD and nephrotic-range proteinuria, which is linked to a faster decline in kidney function [8]. In addition, GLP-1R agonists may act as renal protectors, dampening DKD progression. Interestingly, targeting the inflammatory and fibrotic mediators of DKD, i.e., IL-1, MMP-9, TNF-a, and TGF-β1, may be a useful approach for reversing the progression of DKD [9]. Clinical trials studying specific molecules that target cytokines (i.e., TNF-a, IL-6, IL-1, and IL-18) or kinases (apoptosis signal-regulating kinase 1) are ongoing. Systems biology, including genomics, epigenomics, transcriptomics, and proteomics, guides the identification of novel mediators in human complex diseases, such as DKD and kidney fibrosis [7,10,11], while several compounds are studied in various kidney disease models [12]. The first-line therapeutic options for hypertension have been established for decades [11]. Preclinical studies focusing specifically on the reversal of hypertensive damage within the glomerulus [12] are evolving. Recently, it was found that trimetazidine (TMZ) improved hypertensive nephropathy and decreased kidney injury in mice via modulation of the PHD2/HIF-1α/HO-1 pathway [13], while Rehmannioside A (ReA) reduced fibrosis, inflammation, and subsequent kidney injury, which was associated with the inhibition of the AT1R/MAPK14/IL-17 pathway [14].

Gene expression illustrates the intermediate phenotype between DNA variation and disease phenotypic variation and may identify genetic and environmental effects on cells and tissues [6]. Recent advances in high-throughput genome-wide gene expression studies have contributed to understanding the molecular mechanisms underlying complex features, such as glomerular diseases. For example, in lupus nephritis, the comparison of gene expression variation in peripheral blood cells, synovium, kidneys, and bone marrow between distinct conditions illustrated differences at the transcriptional or post-transcriptional level, leading to distinct molecular pathways, which may be used as targeted therapies [15,16,17,18]. Accordingly, high-throughput genome-wide gene expression studies have characterized the transcriptome of the kidneys and peripheral blood from animal models and patients with glomerulopathies and revealed molecular pathways involved in their pathogenesis [19]. However, gene expression patterns within the glomerulus that are unique to DKD or HTN have not been defined.

Computational systems biology links knowledge-driven experimental data with simulation-based analyses to test biological hypotheses in silico [20]. This strategy constitutes a robust framework to elucidate complex biological mechanisms and identify novel drugs or drugs to be repurposed [6,21]. The Connectivity Map (CMap) project generated for the first time genome-wide gene expression responses from four human cell lines, which were exposed to 1309 FDA-approved drugs and small-molecule compounds across multiple concentrations [6,22]. The NIH Library of Integrated Network-based Cell-Signatures (LINCS) program expanded the CMap project to involve more than one million signatures using the L1000 high-throughput transcriptomic technology. Consequently, it recognized changes in gene expression (pre- and post-treatment) across over 60 human cell lines with more than 20,000 drugs/small-molecule compounds [6,23,24]. Using multivariate methods to compute signatures, the LINCS L1000 Characteristic Direction Signatures Search engine (L1000CDS^2^) evaluated and ranked numerous small-molecule signatures and their paired combinations, which may replicate or counteract the gene expression patterns linked to specific diseases or conditions [6,25].

In this study, using computational systems biology, we identified datasets describing kidney gene expression patterns from patients with DKD or HTN and healthy individuals. By analyzing the kidney gene expression patterns from the identified datasets, we characterized the distinct transcriptional profile associated with kidney disease resulting from diabetes mellitus or hypertension. Differentially expressed genes were functionally annotated using enrichment analysis, leading to the establishment of distinct biological processes and pathways implicated in these diseases. We also inferred networks of transcription factors, protein–protein interactions, and kinases predicted to regulate the expression of the recorded differentially expressed genes. Finally, we identified putative novel drugs or small-molecule compounds that may reverse each disease-specific phenotype, suggesting they should be further tested as potential targets in DKD or HTN.

## 2. Materials and Methods

We used the Nephroseq classic v4 web-based engine to collect gene expression data of human glomeruli from patients with DKD or HTN [20]. NephroSeq (University of Michigan, Ann Arbor, MI, USA) is a curated platform integrating multiple publicly available GEO datasets, and it provides harmonized, comparison-level results rather than raw, dataset-specific outputs [20]. We included datasets generated by Affymetrix Microarray Technology. The keywords “human”, “kidney”, “glomerulus”, “microarray”, and “affymetrix” were used to identify relevant gene expression patterns of diseases. Gene expression patterns from DKD and HTN were compared to those from healthy kidneys. Publicly available kidney gene expression datasets were retrieved from the NCBI Gene Expression Omnibus (GEO): GSE30122 (https://www.ncbi.nlm.nih.gov/geo/query/acc.cgi?acc=GSE30122), GSE69438 (https://www.ncbi.nlm.nih.gov/geo/query/acc.cgi?acc=GSE69438), and the ERCB/KFB cohort GSE47183 (https://www.ncbi.nlm.nih.gov/geo/query/acc.cgi?acc=GSE47183) and GSE47184 (https://www.ncbi.nlm.nih.gov/geo/query/acc.cgi?acc=GSE47184). Control samples were defined according to the original studies and GEO metadata and derived from biopsies of healthy living [26,27,28] or cadaveric [28] transplant donors or the unaffected portion of tumor nephrectomies [27] or biopsies from patients with minimal change disease [28], depending on the dataset. Age and sex were matched between patients and controls across the different studies [26,27,28]. To account for multiple hypothesis testing, false discovery rate (FDR) correction was applied, and statistical significance was defined using q-values, with a threshold of q-value < 0.05. Genes with a q-value < 0.05 were considered significantly differentially expressed, and further analysis was based on these genes [20]. Differential gene expression comparisons were performed with Venny2.1 [29] and InteractiVenn [30] (https://www.interactivenn.net/).

Enrichment analysis of DEGs was conducted using g:Profiler [31] (Institute of Computer Science, University of Tartu, Tartu, Estonia, https://biit.cs.ut.ee/gprofiler/gost, accessed on 25 April 2025). In g:Profiler, Benjamini–Hochberg FDR 0.05 was used as a significance threshold [31]. Transcription factor enrichment analysis, protein–protein interaction network expansion, and kinase enrichment analysis were conducted with the X2K Web engine [32] (Icahn School of Medicine at Mount Sinai/Ma’ayan Lab, New York, NY, USA, https://maayanlab.cloud/X2K/, accessed on 25 April 2025). The L1000CDS^2^ engine (Ma’ayan Laboratory, Icahn School of Medicine at Mount Sinai, New York, NY, USA, https://maayanlab.cloud/L1000CDS2/#/index, accessed on 25 April 2025) was employed to identify compounds capable of reversing the disease-specific gene expression signatures [25]. L1000CDS^2^ provides in silico results based on the LINCS L1000 dataset, which consists primarily of gene expression profiles from chemically/genetically perturbed human cell lines (in vitro). Therefore, our findings represent in vitro/in silico evidence and lack in vivo validation. L1000CDS^2^ engine does not publish conventional sensitivity, specificity, or confidence scores for its genome-wide matching results, like a ROC curve or *p*-value thresholding system. Moreover, there is an absence of a comprehensive study that compares L1000CDS^2^ outputs with datasets containing known positive and negative associations, in a way that generates ROC curves to assess the tool’s performance [25]. To investigate potential disease targets, mechanisms of action, adverse effects, and FDA approval status of the drugs or small molecules, we employed the Large-Scale Visualization of Drug-induced Transcriptomic signatures (L1000FWD) tool [33], the DrugBank resource [34,35] (https://go.drugbank.com/), and the FDA website [36] (https://www.fda.gov/).

## 3. Results

### 3.1. Computational Systems Biology Approaches Reveal Gene Signatures Specific for Diabetic and Hypertensive Kidney Disease

**Figure 1:**
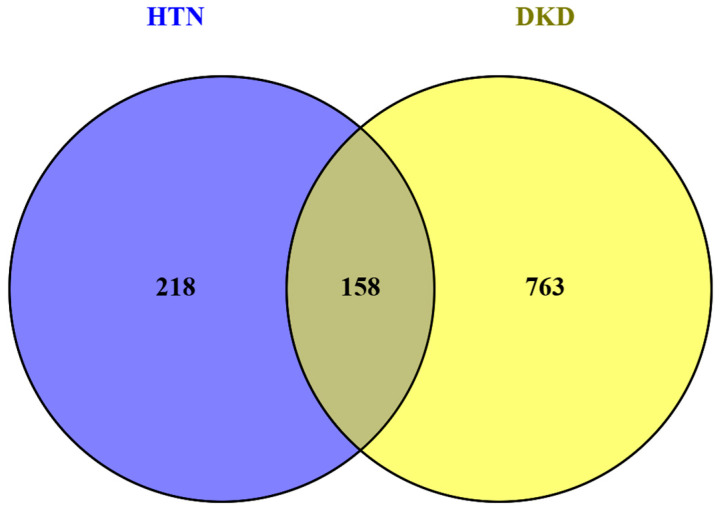
DEGs in HTN, DKD, and common.

Datasets comparing kidney gene expression from patients with DKD (a total of 38 patients) [26,27,28] or HTN (20 patients) [26] vs. healthy individuals (a total of 50) were retrieved. To identify the unique transcriptional landscape of each nephropathy, gene expression patterns between the yielded datasets were compared. Differentially expressed genes (DEGs) uniquely detected in one disease, but not in the other, were used to define the disease-specific gene signature. A total of 921 DEGs were identified within kidneys from DKD versus healthy (434 in glomeruli, 525 in tubulointerstitium, 463 overexpressed, and 463 underexpressed), and 376 DEGs were identified within kidneys from HTN versus healthy (237 in glomeruli, 155 in tubulointerstitium, 151 overexpressed, and 225 underexpressed). Accordingly, 763 and 218 DEGs defined the DKD-specific and HTN-specific gene signatures, respectively, implying a distinct pathogenic intra-tissue involvement highly specific to the respective disease. A total of 158 DEGs were common between DKD and HTN (Figure 1). These data demonstrate that, in DKD and HTN, gene expression profiles within kidneys are deregulated in a disease-specific manner, whereas common DEGs can be found in both diseases. Further experimental validation is required to confirm differential transcripts and their functional roles in the kidneys.

### 3.2. Unique Biological Processes and Pathways Are Implicated in Diabetic and Hypertensive Glomerular Diseases

**Figure 2:**
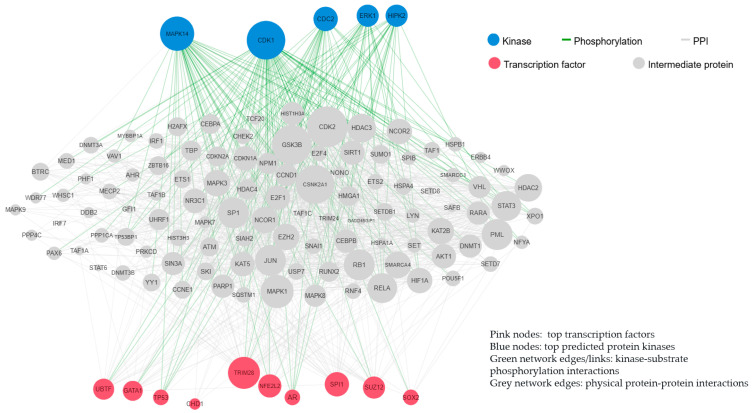
Protein–protein interactions and kinase–substrate phosphorylation interactions in DKD.

The DKD-specific gene signature was significantly enriched for GO biological processes linked to interleukin-4 and interleukin-13 signaling and interferon gamma signaling, and was regulated by the transcription factors TP53 and NFE2L2, which generated protein–protein interactions regulated by ERK1 and CDK1 kinases (Figure 2).

**Figure 3:**
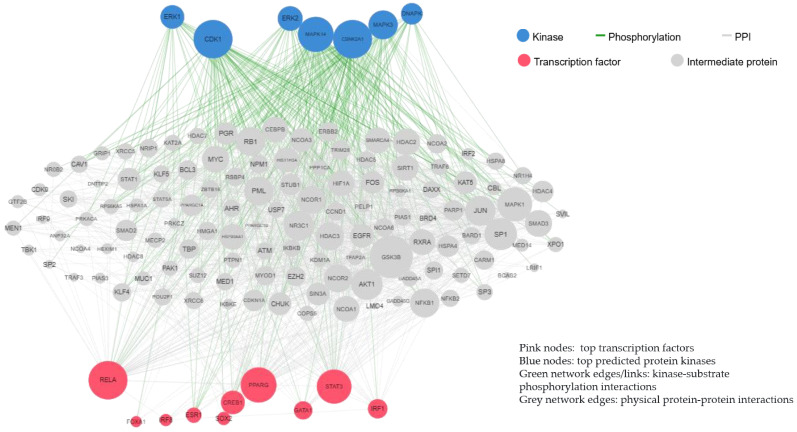
Protein–protein interactions and kinase–substrate phosphorylation interactions in HTN.

The HTN-specific gene signature showed significant enrichment in Gene Ontology (GO) biological processes linked to Th17 cell differentiation and Th1 and Th2 cell differentiation, which was regulated by the transcription factors PPARG and STAT3, which generated protein–protein interactions regulated by MAPK3 και CDK1 kinases (Figure 3). 

**Figure 4:**
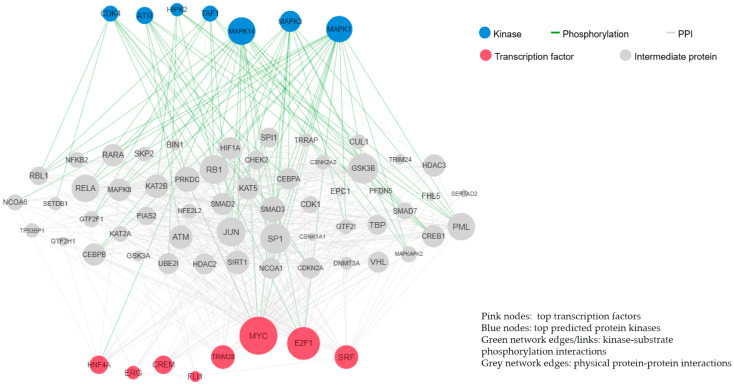
Common protein–protein interactions and kinase–substrate phosphorylation interactions in DKD and HTN.

The common DEGs between DKD and HTN were enriched in the AGE-RAGE signaling pathway in diabetic complications and the PI3K-Akt signaling pathway, which were regulated by the transcription factors MYC and E2F1, which generated protein–protein interactions regulated by MAPK3 and MAPK14 kinases (Figure 4). Collectively, these data are demonstrated as followed.

The involvement of unique biological processes and pathways in distinct kidney diseases suggests that these processes and pathways may represent potential novel disease-specific therapeutic targets. Simultaneously, the common pathways highlight the possibility that new potential therapeutic targets may be effective against both DKD and HTN.

### 3.3. Novel Drugs or Small-Molecule Compounds That May Reverse Kidney-Specific Gene Signatures in Diabetic and Hypertensive Kidney Disease

To identify potential novel drugs or small-molecule compounds capable of reversing disease-specific kidney phenotypes, the L1000CDS^2^ platform was queried using upregulated and downregulated genes from each disease to prioritize inversely correlated transcriptional signatures. The top 50 compounds predicted to reverse each disease-associated signature were identified. To determine kidney disease-specific therapeutic candidates, the top-ranked drug signatures were compared between DKD and HTN. Compounds predicted to reverse only one disease-specific gene signature, but not the other, were defined as disease-specific drug candidates. Thus, 20 drugs/small-molecule compounds were predicted to reverse the DKD-specific gene signature, while 11 drugs/compounds were predicted to reverse the HTN-specific gene signature (Table 1).

In addition, 39 drugs were able to reverse both kidney diseases (Table 2).

The potential mechanism of action of identified drugs and small molecules, the disease they target, their possible side effects, and their FDA approval are described in Table 3.

Collectively, these data reveal novel, not previously described, drugs and small-molecule compounds that may reverse the phenotype of DKD and HTN in a disease-specific manner. The majority of them are investigational drugs and not FDA-approved. However, some of them are known nephrotoxic agents. For example, 5-azacytidine causes renal tubular dysfunction, which may be mitigated via adequate hydration and dose modification.

In the following section, we aim to highlight the need for a better understanding of the pathophysiology behind these diseases and the development of novel drugs, which specifically target the DEGs expressed in each nephropathy. However, further investigation is required to assess the therapeutic implications of our findings.

## 4. Discussion

Herein, we discovered gene expression datasets within kidneys from patients with diabetic (DKD) or hypertensive (HTN) kidney disease compared to healthy individuals using computational systems biology. This comparison was of gene expression patterns defined as DKD-specific and HTN-specific, as well as the common gene signatures. Functional enrichment analysis of differentially expressed genes identified distinct biological processes and pathways implicated in each kidney disease, as well as common pathways implicated in both kidney diseases. Moreover, we uncovered novel, not previously determined, drugs and small-molecule compounds that may reverse the phenotype of these kidney diseases in a kidney disease-specific and non-specific manner. The validation of putative targets is ongoing and was beyond the scope of this manuscript.

Inflammation and fibrosis play a key role in the pathogenesis of both DKD and HTN [3,37], a finding also confirmed by our study. Molecular alterations occur from the beginning of the disease, highlighting the importance of our findings in altering their natural course. Specific targeting of the implicated biologic pathways may provide a new strategy for managing these problems.

The already established therapeutic options for DKD include RAS inhibition, while sodium-glucose cotransporter-2 (SGLT2) inhibitors, GLP-1 receptor agonists, and finerenone constitute the newest drugs used in clinical practice for kidney protection [7]. The first-line therapeutic options for hypertension include thiazide-type diuretics, RAS inhibitors, and calcium channel blockers (CCBs) [11]. Preclinical studies have shown anti-inflammatory properties of RAS inhibitors, finerenone, SGLT2 inhibitors, and GLP1 receptor agonists in diabetic kidney disease [7]. However, improvement of inflammation may be a secondary result of the other beneficial outcomes, such as improvement in glomerular hyperfiltration and albuminuria [7]. Undoubtedly, the already established therapy is effective, as, for instance, SGLT2 inhibitors were associated with a reduced risk of major adverse cardiovascular events, hospitalization for heart failure, and kidney outcomes in patients with diabetes [38]. The limitations of current therapies lie in the adverse events, polypharmacy, or modest response, for instance, to antihypertensive drugs in patients with resistant hypertension. Moreover, SGLT2 and DPP-4 inhibitors leave a substantial risk for progression to end-stage kidney disease in patients with diabetes [39,40]. Further research is needed to fully elucidate which pathways participate in the development of DKD and HTN. Expanding our knowledge of these pathways will enhance the development of causal and targeted therapies, which specifically act on the glomerulus, reverse the implicated biological pathways, and facilitate the timely prevention of kidney injury. Nano-particle delivery systems could aid in the transport of these drugs to the kidney [41]. Safety requirements, including initial considerations of pharmacokinetics and toxicology, before progression to human studies, as well as discussion of which candidates are more likely to be translated based on existing experience or mechanistic alignment, are needed. It is important to note that this study does not aim to undermine established therapeutic strategies; rather, it seeks to broaden the spectrum of available treatment options for these kidney diseases.

**Figure 5:**
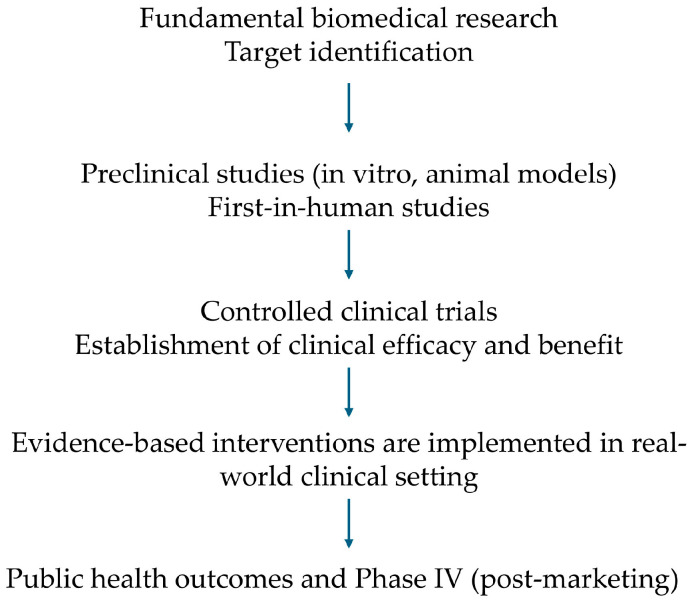
Clinical translation framework.

Using kidney disease-specific gene signatures, we discovered through computational systems biology approaches, novel agents or drugs to be repurposed in a manner specific to each disease. Specifically, we employed the L1000CDS^2^ engine [25] and prioritized the top 50 molecules predicted to reverse upregulated and downregulated DEGs of each kidney disease, as well as the common DEGs between DKD and HTN. Drug signatures of both diseases were compared, resulting in the identification of nephropathy-specific and non-specific drugs, uncovering underlying intra-tissue molecular mechanisms. Each drug was associated with the inhibition or modulation of a specific biological pathway or process, implying a possible role of the respective pathway in the pathogenesis of DKD and HTN. Previous trials targeting single cytokines, such as IL-6 or TNF-α, likely failed because these molecules are downstream markers in complex immune pathways and do not drive disease in all patients. Our analysis instead identifies broader pathway modules, such as IL-4/IL-13, which reflect upstream immune activation, organ damage, and patient-specific biology. This approach better reflects disease diversity and clarifies why single-target interventions were of limited success. A clinical translation framework is provided in Figure 5, maximizing the translational impact of our findings.

Among other drugs, parthenolide, an inhibitor of the proto-oncogene c-Rel, and vorinostat, an inhibitor of the enzymatic activity of histone deacetylases HDAC1, HDAC2, and HDAC3, reversed the common DEGs between DKD and HTN. TW 37 reversed the DEGs of HTN and is studied for its therapeutic potential in kidney diseases, as it inhibits KIM-1 (Kidney Injury Molecule-1)-mediated endocytosis, resulting in a reduction in NF-κB and IL-1β, inhibition of the NF-κB-NLRP3-IL-1β pathway, and a subsequent decrease in fibrosis and inflammation [42]. Geldanamycin, a heat shock protein HSP 90-alpha inhibitor and endoplasmin inhibitor, specifically reverses the diabetic nephropathy-specific gene signature and has been associated with the reversal of kidney function and improvement of the glomerular and tubular damage by a high-fat diet in db/db mice [43]. Through computational analysis, we prioritized drugs as disease-specific and non-specific agents for each kidney disease. Further investigation is required to unravel the therapeutic implications of our findings.

Collectively, we defined the diabetic nephropathy-specific and hypertensive nephropathy-specific gene signatures within human kidneys and identified distinct biological processes and pathways associated with each disease through computational systems biology approaches. We also discovered novel, not previously identified, drugs and small-molecule compounds that may reverse the phenotype of these kidney diseases in a kidney disease-specific and non-specific manner. However, experimental validation of the differential transcripts and their functionality is further required, in addition to future studies defining the pathogenic and therapeutic implications of our results.

A limitation of our study is the inability to perform batch effect assessment and correction using standard methods, as this study is based on integrated, publicly available transcriptomic data obtained from the NephroSeq platform. Albeit NephroSeq applies internal normalization procedures, access to raw expression data and complete sample-level metadata is limited. Moreover, detailed clinical information at the individual sample level, i.e., disease severity, medication use, comorbidities, and ethnicity, was not available, restricting the ability to evaluate potential confounding factors. For the same reason, a formal sensitivity analysis examining the stability of the identified DEGs and downstream drug predictions upon exclusion of individual datasets could not be conducted.

Another limitation is the inability to report detailed, dataset-specific information (e.g., individual GSE accession numbers, sample sizes, platforms, and ethical approvals), as such metadata that are not fully accessible within the NephroSeq framework.

Regarding external validation of the data from L1000CDS^2^, there is an absence of a comprehensive study that compares L1000CDS^2^ outputs with datasets containing known positive and negative associations, in a way that generates ROC curves to assess the performance of L1000CDS^2^.

These limitations should be considered when interpreting the results, and future studies using fully accessible raw data and comprehensive clinical annotation are necessary to validate the robustness of the findings.

## 5. Conclusions

In this study, we used an integrative computational systems biology framework to compare the molecular profiles of diabetic kidney disease (DKD) and hypertensive nephropathy (HTN). By integrating multiple independent transcriptomic datasets and stratifying gene expression changes across glomeruli and tubulointerstitium, we identified robust disease-specific and shared gene signatures that highlight distinct and common pathogenic mechanisms behind these diseases. This comparative strategy represents a key innovation of the study, enabling the molecular discrimination between two clinically related but mechanistically distinct nephropathies.

Beyond differential gene expression analysis, we integrated functional enrichment analysis, transcription factor regulation, and protein–protein interaction networks to construct multilevel regulatory maps for each disease. As a result, unique and convergent immune- and inflammation-related pathways in DKD and HTN were detected, providing a mechanistic insight that extends beyond single-gene observations. Importantly, we associated disease-specific gene signatures with large-scale perturbational transcriptomic data to predict candidate drugs and small-molecule compounds, which are able to reverse pathological gene expression patterns. This in silico drug repurposing strategy constitutes a major innovative contribution, as it bridges molecular disease signatures with actionable therapeutic hypotheses.

Collectively, our findings demonstrate that DKD and HTN are characterized by distinct and partially overlapping molecular pathways that can be systematically resolved using computational systems biology. These results also underscore the need for future studies to validate the predicted drug candidates experimentally and clinically, to incorporate additional multi-omics layers, and to assess disease heterogeneity across various patient populations. Such efforts will be essential to translate these molecular insights into effective, personalized therapeutic options. As a result, extending this framework through longitudinal data, functional validation, and clinical trials will be critical for advancing precision medicine approaches in kidney disease.

## Figures and Tables

**Table 1:** Drugs/small-molecule compounds reversing DKD-specific and HTN-specific gene signatures.

Rank	Score	Perturbation	Cell-Line	Dose	Time
		**Hypertensive Nephropathy**			
13	0.0728	TW 37	PC3	10.0 µm	24.0 h
15	0.0728	BRD-K25737009	SW620	40.0 µm	6.0 h
16	0.0728	BRD-K58306044	A375	10.0 µm	6.0 h
18	0.0701	BRD-A18763547	A375	10.0 µm	24.0 h
27	0.0701	BRD-A82371568	A375	10.0 µm	6.0 h
29	0.0701	withaferin-a	BT20	3.33 µm	24 h
36	0.0674	perhexiline maleate	HT115	10.0 µm	6.0 h
41	0.0674	BRD-K56411643	VCAP	10.0 µm	6.0 h
45	0.0674	BRD-K19166598	MCF7	10.0 µm	24.0 h
46	0.0674	BRD-K03109492	A375	10.0 µm	6.0 h
		**Diabetic Nephropathy**			
22	0.0370	triamcinolone acetonide	A549	10.0 µm	6.0 h
33	0.0348	BRD-K52075040	A375	44.4 µm	24.0 h
34	0.0348	BRD-K81709173	A549	10.0 µm	6.0 h
40	0.0337	BI 2536	VCAP	10.0 µm	24.0 h
41	0.0337	BI-2536	A549	10 µm	24 h
42	0.0337	pracinostat	A549	10 µm	24 h
44	0.0325	anisomycin	HCC515	10.0 µm	24.0 h
45	0.0325	curcubitacin I	A375	10.0 µm	24.0 h
46	0.0325	528116.cdx	A375	0.09 µm	24.0 h
47	0.0325	Chemistry 2804	A375	10.0 µm	24.0 h
49	0.0325	piperlongumine (HPLC)	PC3	10.0 µm	24.0 h

**Table 2:** Drugs/small-molecule compounds reversing both kidney diseases.

Rank	Score	Perturbation	Cell-Line	Dose	Time
1	0.1274	vorinostat	PC3	10 µm	24 h
2	0.1210	cercosporin	A375	10.0 µm	24.0 h
3	0.1210	trichostatin A	PC3	10.0 µm	24.0 h
4	0.1210	dexamethasone	A549	10.0 µm	6.0 h
5	0.1210	BRD-K60640630	A549	10.0 µm	24.0 h
6	0.1146	triamcinolone acetonide	A549	10.0 µm	6.0 h
7	0.1146	narciclasine	A375	10.0 µm	24.0 h
8	0.1146	niclosamide	A375	10.0 µm	24.0 h
10	0.1146	BRD-K99633092	PC3	10.0 µm	6.0 h
11	0.1146	LDN-193189	SKBR3	10 µm	3 h
12	0.1146	QL-XII-47	MDAMB231	3.33 µm	3 h
15	0.1083	manumycin A	PC3	10.0 µm	24.0 h
16	0.1083	15-Deoxy-Δ12,14-prostaglandin J2	A375	10.0 µm	24.0 h
17	0.1083	Ro 28-1675	A549	160.0 µm	6.0 h
18	0.1083	DG-041	A549	40.0 µm	6.0 h
20	0.1083	COT-10b	HT115	44.4 µm	6.0 h
21	0.1083	5-azacytidine	A375	10.0 µm	6.0 h
25	0.1019	proscillaridin A	HA1E	10.0 µm	6.0 h
27	0.1019	cycloheximide	PC3	10.0 µm	24.0 h
28	0.1019	L-690,330	A549	10.0 µm	6.0 h
29	0.1019	Akt inhibitor IV	HT115	10.0 µm	6.0 h
30	0.1019	YM-155	MDST8	0.31 µm	6.0 h
32	0.1019	salermide	PC3	120.0 µm	24.0 h
33	0.1019	emetine hydrochloride	A549	10.0 µm	6.0 h
34	0.1019	desoximetasone	HCC515	10.0 µm	6.0 h
35	0.1019	BRD-K92317137	HEPG2	10.0 µm	6.0 h
36	0.1019	V4877	SKB	10.0 µm	24.0 h
37	0.1019	BRD-A58564983	A375	10.0 µm	6.0 h
38	0.1019	BRD-A26095496	A549	10.0 µm	24.0 h
39	0.1019	BRD-A63894585	A549	10.0 µm	24.0 h
45	0.0955	clocortolone pivalate	HCC515	10.0 µm	24.0 h
46	0.0955	betamethasone	HCC515	10.0 µm	6.0 h
47	0.0955	alclometazone dipropionate	HCC515	10.0 µm	6.0 h
48	0.0955	triamcinolone diacetate	A549	10.0 µm	24.0 h
49	0.0955	triamcinolone	A549	10.0 µm	24.0 h
4	0.0809	BRD-K84203638	A375	10.0 µm	24.0 h
32	0.0674	HDAC6 inhibitor ISOX	A375	10.0 µm	24.0 h
21	0.0701	parthenolide	A375	10.0 µm	24.0 h
8	0.0755	BRD-K04853698	MCF7	10.0 µm	6.0 h

**Table 3:** Mechanism of action of identified drugs/small-molecule compounds, FDA approval, disease-of-target, and possible side effects.

Drug	Mechanism of Action	FDA Approval	Disease Target	Side Effects
BRD-K19295594	binding to Bcl-2 and Bcl-xL	YES	infections in the ear canal	reproductive system, heart, liver, membranes
TW 37	inhibitor of Bcl-2	NO	N/A	N/A
Perhexiline maleate	coronary vasodilator	NO	severe angina pectoris	Neuropathy, hepatitis
Geldanamycin	heat shock protein HSP 90-alpha inhibitor, endoplasmin inhibitor	NO	antimicrobial activity against many Gram-positive and some Gram-negative bacteria, antiviral activity, antineoplastic activity	hepatotoxicity, gastrointestinal issues, fatigue, headache
BI-2536	serine/threonine-protein kinase PLK1 inhibitor	NO	Under investigation for advanced or metastatic non-small cell lung cancer	fatigue, leukopenia, nausea
Pracinostat	HDAC inhibitor	NO	hematological and solid tumors	hematologic toxicities, fatigue, and gastrointestinal issues
Vorinostat	histone deacetylase (HDAC) inhibitor	YES	cutaneous T- cell lymphoma (CTCL)	Hepatotoxicity
Parthenolide	proto-oncogene c-Rel inhibitor, transcription factor RelB inhibitor	NO	N/A	GI symptoms, allergic contact dermatitis, withdrawal symptoms
Trichostatin A	histone deacetylase inhibitor	YES	cutaneous T cell lymphoma (CTCL)	acute toxicity, skin/eye/respiratory irritation
Dexamethasone	decreased vasodilation and permeability of capillaries, decreased leukocyte migration to sites of inflammation	YES	bronchial asthma, as well as endocrine and rheumatic disorders	cataract, mood changes, hypertension, hyperlipidemia, peptic ulcer, pancreatitis, myopathy, osteoporosis
BRD-K60640630	inhibition of mast cells, eosinophils, basophils, and lymphocytes, inhibition of histamine, leukotrienes, and cytokine	YES	asthma, rhinitis, and certain skin conditions	Hypercorticism, adrenal suppression
Niclosamide	DNA antagonist	YES	tapeworm infections	nausea, vomiting, diarrhea and abdominal discomfort
Clocortolone pivalate	induction of phospholipase A2 inhibitory proteins	YES	inflammatory and pruritic scalp dermatoses	thinning of skin and suppression of adrenal cortex
Desoximetasone	induction of phospholipase A2 inhibitory proteins	YES	inflammatory and pruritic corticosteroid- responsive dermatoses	Skin thinning and suppression of adrenal cortex
5-azacytidine	pyrimidine nucleoside analog	YES	certain subtypes of myelodysplastic syndrome	diarrhea, nausea, and vomiting
Betamethasone	inhibition of neutrophil apoptosis and demargination, NF-Kappa B, phospholipase A2 and promotion of anti-inflammatory genes, like interleukin-10	YES	disorders of skin, hormones, digestive system and blood	cataracts, hypertension, water retention, hyperlipidemia, peptic ulcer, myopathy, osteoporosis, mood changes, psychosis, dermal atrophy, allergy, acne
Triamcinolone	inhibition of phospholipase A2 on cell membranes	YES	allergic rhinitis, multiple sclerosis exacerbations, osteoarthritic knee pain, corticosteroid responsive dermatoses	Cushing’s syndrome

## Data Availability

The original contributions presented in this study are included in the article. Further inquiries can be directed to the corresponding author.

## Abbreviations

The following abbreviations are used in this manuscript:

DKD	Diabetic kidney disease
HTN	Hypertensive nephropathy
RAS	Renin–angiotensin system
MRA	Mineralocorticoid receptor antagonist
SGLT2-i	Sodium-glucose cotransporter 2-inhibition
DPP-4	Dipeptidyl peptidase-4
